# Antibiotic Use in the Community in Spain: A National Surveillance System Within the Framework of the Spanish Action Plan on Antimicrobial Resistance

**DOI:** 10.3390/antibiotics14111071

**Published:** 2025-10-24

**Authors:** Rocío Fernández-Urrusuno, Carmen Marina Meseguer-Barros, María García-Gil, Itxasne Lekue-Alkorta, María Belén Pina-Gadea, María Ana Prado-Prieto, Natalia Alzueta-Isturiz, Lucía Jamart-Sánchez, Laura Villar-Gómara, Antonio López-Navas

**Affiliations:** 1 Service of Pharmacy, Sevilla Primary Health Care District, Av. Jerez, 41013 Sevilla, Spain; 2 Service of Pharmacy, Ouest Primary Health Care Area, Madrid Health Service, Madrid C/Alonso Cano, Num 8, Mostoles, 28933 Madrid, Spain; 3 Service of Primary Care Pharmacy, Sagunto Health Care Department, Comunidad Valenciana, Avda. Ramón y Cajal s/n, 46520 Sagunto, Spain; 4 Primary Care Pharmacy Service, Ezkerraldea-Enkarterri-Cruces Integrated Health Organization, Osakidetza, Plaza Cruces s/n, 48903 Barakaldo, Spain; 5 Service of Primary Care Pharmacy, Zaragoza II Sector, Aragón Health Service, Zaragoza, C/Condes de Aragón 30, 50009 Zaragoza, Spain; 6 Service of Pharmacy, East Valladolid Primary Health Care, Castilla y Leon Health, Valladolid, Castilla y León, Cardenal Torquemada nº 54, 47010 Valladolid, Spain; 7 Medicines Advice and Information Service, Navarra Health Service, Pamplona, Plaza de la Paz s/n, 31002 Pamplona, Spain; 8 Coordination Unit of the Spanish National Action Plan on Antimicrobial Resistance, Spanish Agency of Medicines and Medical Devices, Calle Campezo 1, Edificio 8, 28022 Madrid, Spain

**Keywords:** antibiotics, primary healthcare, database, community, antimicrobial stewardship, surveillance system, population base, antimicrobial resistance, outpatient use

## Abstract

**Background:** Antimicrobial resistance (AMR) remains a critical major public health challenge, largely driven by the inappropriate use of antibiotics in the community. In Spain, the National Action Plan on AMR (PRAN) emphasizes the need for robust surveillance systems based on standardized indicators and high-quality data sources. **Objective:** This study aimed to evaluate the feasibility of calculating PRAN prescribing indicators using the National Electronic Database for Pharmacoepidemiological Research in Primary Care (BIFAP) and to validate BIFAP as a data source for national antimicrobial prescribing surveillance. **Methods:** A population-based cross-sectional study was conducted using 2018 data from 9.4 million individuals. **Results:** Overall, 23.3% received at least one antibiotic prescription during the year, with an average of 1.8 treatments per patient. First-line recommended antibiotics represented 26.5% of total dispensed defined daily doses. Notable age-related variability in prescribing patterns was observed: children predominantly received first-line narrow-spectrum antibiotics, whereas older adults were more frequently prescribed broad-spectrum agents. **Discusion:** BIFAP-based indicators closely aligned with PRAN data while allowing for the calculation of additional metrics, such as prevalence of use, treatments per patient-year, and variations by age and sex. The findings underscore the importance of patient-level monitoring to identify demographic-age-specific priorities for targeted interventions aimed at optimizing antibiotic use in Primary Care. **Conclusions:** This study confirms the feasibility of using BIFAP to strengthen antibiotic consumption monitoring and policy evaluation efforts in Spain.

## 1. Introduction

Antimicrobial resistance (AMR) is widely recognized as a major threat to public health [1,2]. Infectious diseases remain highly prevalent in the community, where nearly two-thirds of patients consulting for infectious conditions receive antibiotic therapy [3]. Consequently, between 15 and 30% of the population in the community are prescribed antibiotics at least once a year, with Primary Care accounting for the majority of these prescriptions [4,5,6]. The inappropriate use of antibiotics in the community not only contributes to the development of AMR but also increases the likelihood of preventable drug-related adverse events, demanding urgent interventions [7]. Thus, the implementation of effective interventions in Primary Care, preferably as a part of comprehensive Antimicrobial Stewardship Programs (ASPs), has become an increasing priority for Health Services and policymakers [8,9]. A key component of ASPs is the availability of robust prescribing indicators that enable the evaluation and understanding of antibiotic use patterns. Analyzing antibiotic consumption is essential to guide both healthcare professionals and policymakers in identifying areas for improvement and assessing the impact of interventions [10].

In Spain, the National Action Plan on AMR (PRAN), coordinated by the Spanish Agency of Medicines and Medical Devices (AEMPS), has developed a set of specific indicators to enable more accurate monitoring and analysis of antibiotic prescribing in the community [11]. These indicators are tailored to the Spanish context and aligned with the recommendations of the National Antimicrobial Therapeutic Guide [12]. In recent years, the PRAN has established a robust system for monitoring antibiotic consumption, providing aggregated data at both regional and national levels through its website [13]. However, the current PRAN prioritizes enhancing the monitoring of antibiotic use by developing new indicators and conducting more detailed data analysis. These efforts aim to improve the understanding of the clinical and epidemiological situation in Spain [14]. On the other hand, PRAN promotes the use of the National Electronic Database for Pharmacoepidemiological Research in Primary Care (BIFAP) [15] as a data source to provide a community-level antibiotic surveillance system [14].

The objective of this study was to expand the current scope of PRAN surveillance system in order to enhance the quality of antibiotic consumption monitoring in the community in Spain. Specifically, the study aimed to: (a) assess the feasibility of PRAN indicators using BIFAP as the data source, (b) obtain more detailed and granular data on community antibiotic consumption, and (c) determine which indicators could be calculated from BIFAP to support its use as a national surveillance system on antibiotic consumption in the community.

## 2. Results

### 2.1. Study Population

Among the 9,390,253 individuals included in the BIFAP database during the study period, a total of 2,191,582 (23.3%) received at least one course of systemic antibacterial agents. These patients accounted for 3,981,672 antibiotic prescriptions.

Baseline characteristics of the study population are summarized in Table 1. The median age was 47 years. Around 85% of patients were adults and 58% were female. BIFAP enabled the collection of other relevant clinical information, including comorbidities and concomitant treatments. Overall, 67.6% of patients had at least one comorbidity; 46% were current or former smokers, and 30% reported alcohol consumption. The prevalence of comorbidities was notably higher among adults (73.5%) compared to pediatric patients (33.9%). As a median, adult patients had two underlying chronic conditions, while pediatric patients had one. Mortality during the study affected less than 1% of patients.

Regarding concomitant treatments, 88.4% of adults and 78.8% of pediatric patients were receiving other medications while taking the prescribed antibiotics. The median number of concomitant treatments was three in adults and two in children. Among adults, most common prescribed concomitant drugs were analgesics, anti-inflammatory agents, proton-pump inhibitors, antihypertensives, benzodiazepines and benzodiazepine related drugs, antiasthmatics and bronchodilators, lipid-lowering agents, antihistamines, and inhaled corticosteroids. Among pediatric patients, the most frequent concomitant medications were anti-inflammatory agents, analgesics, inhaled corticosteroids, antiasthmatic and bronchodilators, and antihistamines.

### 2.2. Antibiotic Use by BIFAP Population

#### 2.2.1. Quantitative Indicators (Table 2)

Antibiotic use in the BIFAP population, expressed as defined daily doses (DDD) per 1000 inhabitants per day (DID), was 10.98. Consumption rates were higher among women and older adults, particularly those aged 65 years and above, with the highest rates observed in individuals over 75 (7 DID and 9.4 percentage points higher than those of adults aged 15–64 years). A total of 23.3% of the BIFAP population received at least one course of antibiotics during the year, with an average of 1.8 treatments per patient per year. The prevalence of antibiotic use was higher among women, with no notable differences in average use between children and adults. Among children, the youngest age group showed the highest prevalence, with one in three children aged 0–4 years receiving antibiotics. In the adult population, antibiotic use increased with age, exceeding 30% among individuals over 75 years.

**Table 2:** Consumption of reimbursed antibiotics (J01) in the community by BIFAP population. Quantitative indicators.

Indicators		Result (Rate, %) [95%CI]	Absolute Difference [95%CI]
Indicator 1. Consumption rates of antibiotics for systemic use (J01) (DID: DDD/1000 inhabitants and days) ^#^	Total	10.98 [10.98–10.99]	
	Men	9.76 [9.75–9.76]	Reference
	Women	12.15 [12.15–12.15]	−2.39 * [−2.40–−2.38]
	Children (<15 years)	7.02 [7.01–7.03]	Reference
	Adults (≥15 years)	11.67 [11.67–11.67]	−4.65 * [−4.66–−4.64]
	0–4 years	7.97 [7.96—7.99]	Reference
	5–9 years	6.98 [6.96–6.99]	−0.99 * [−1.02–−0.96]
	10–14 years	6.11 [6.10–6.13]	−1.86 * [−1.89–−1.83]
	15–64 years	10.27 [10.26–10.27]	Reference
	65–74 years	14.14 [14.13–14.15]	3.87 * [3.84–3.90]
	≥75 years	17.36 [17.35–17.37]	7.09 * [7.06–7.12]
Indicator 2. Prevalence of antibiotic use (%)	Total	23.34 [23.31–23.37]	
	Men	19.96 [19.92–19.99]	Reference
	Women	26.55 [26.51–26.59]	6.59 * [6.57–6.61]
	Children (<15 years)	23.31 [23.24–23.38]	Reference
	Adults (≥15 years)	23.34 [23.31–23.37]	0.03 ^NS^ [−0.01–0.07]
	0–4 years	29.13 [29.00–29.26]	Reference
	5–9 years	23.83 [23.70–23.95]	−5.30 * [−5.38–−5.22]
	10–14 years	16.91 [16.80–17.01]	−12.22 * [−12.30–−12.14]
	15–64 years	21.44 [21.40–21.47]	Reference
	65–74 years	26.97 [26.88–27.06]	5.53 * [5.47–5.59]
	≥75 years	30.86 [30.77–30.95]	9.42 * [9.36–9.48]
Indicator 3. Total number of antibiotics dispensations per patient-year ^#^	Total	1.82 [1.81–1.82]	
	Men	1.74 [1.73–1.75]	Reference
	Women	1.87 [1.86–1.88]	0.13 * [0.12–0.14]
	Children (<15 years)	1.81 [1.80–1.83]	Reference
	Adults (≥15 years)	1.82 [1.81–1.82]	0.01 ^NS^ [0.00–0.02]
	0–4 years	2.08 [2.05–2.10]	Reference
	5–9 years	1.73 [1.70–1.75]	−0.35 * [−0.37–−0.33]
	10–14 years	1.48 [1.45–1.50]	−0.60 * [−0.62–−0.58]
	15–64 years	1.69 [1.68–1.69]	Reference
	65–74 years	1.96 [1.94–1.97]	0.27 * [0.25–0.29]
	≥75 years	2.22 [2.20–2.23]	0.53 * [0.51–0.55]

^#^ Data are represented as the mean with 95%CI in square brackets. * Indicates statistical significance; *p* < 0.001; ^NS^: Not significant. Student’s *t*-test and ANOVA for indicators 1 and 3; Chi-square test for indicator 2.

#### 2.2.2. Qualitative Indicators (Table 3)

First-line antibiotics recommended for the treatment of community-acquired infections by the national guide [12] accounted for 26.5% of the total dispensed DDD and 33.9% of the dispensed packages. These data varied substantially across age groups: in children they represented 50.5% of total use, compared to 24.0% in adults, with the lowest proportion observed in individuals aged ≥75 years (18.0%). First-line antibiotics, such as amoxicillin and fosfomycin, accounted for 22.1% and 2.6% of dispensed DDD, respectively. In terms of packages, amoxicillin represented 20.5% of dispensations, while fosfomycin accounted for 10.5%. When analyzed by age group, amoxicillin was more commonly prescribed to pediatric patients regardless of sex. Fosfomycin showed a sex- and age-dependent pattern, being predominantly prescribed to older women. Beta-lactamase-sensitive penicillins represented a marginal proportion of antibiotic use in Spain, accounting for 0.6% of all dispensed antibiotics. Their use was almost limited to the pediatric population.

In contrast, second-line and third-line antibiotics such as amoxicillin and beta-lactamase inhibitor, macrolides, fluoroquinolones, and third-generation cephalosporins represented 56.5% of dispensed DDD and 54.5% of packages.

**Table 3:** Consumption of reimbursed antibiotics (J01) in the community by BIFAP population. Qualitative indicators.

Indicator		Results Using DDD as Measurement Unit (%) [95%CI]	Absolute Difference [95%CI]	Results Using Packages as Measurement Unit (%) [95%CI]	Absolute Difference [95%CI]
Indicator 4. Percentage of first-line antibiotics ^#^ (%)	Total	26.52 [26.51–26.53]		33.85 [33.81–33.90]	
	Men	24.46 [24.44–24.48]	Reference	28.54 [28.47–28.61]	Reference
	Women	28.09 [28.08–28.11]	3.63 * [3.60–3.66]	37.46 [37.40–37.52]	8.92 * [8.88–8.96]
	Children (<15 years)	50.49 [50.44–50.54]	Reference	57.59 [57.47–57.71]	Reference
	Adults (≥15 years)	24.02 [24.01–24.03]	−26.47 * [−26.53–−26.41]	29.29 [29.24–29.34]	−28.30 * [−28.34–−28.26]
	0–4 years	54.42 [54.33–54.50]	Reference	60.84 [60.68–60.01]	Reference
	5–9 years	51.04 [50.95–51.13]	−3.38 * [−3.57–−3.19]	59.08 [58.88–59.28]	−1.76 * [−1.84–−1.68]
	10–14 years	44.68 [44.59–44.78]	−9.74 * [−9.94–−9.54]	46.54 [46.27–46.82]	−14.30 * [−14.38–−14.22]
	15–64 years	26.26 [26.24–26.28]	Reference	31.13 [31.07–31.19]	Reference
	65–74 years	21.98 [21.94–22.02]	−4.28 * [–4.43–−4.14]	26.50 [26.38–26.62]	−4.63 * [−4.69–−4.57]
	≥75 years	18.02 [17.99–18.05]	−8.24 * [−8.40–−8.09]	25.94 [25.85–26.04]	−5.19 * [−5.25–−5.13]
Indicator 5. Percentage of beta-lactamase sensitive penicillins (%)	Total	0.59 [0.59–0.59]		1.52 [1.51–1.53]	
	Men	0.58 [0.58–0.59]	Reference	1.69 [1.67–1.91]	Reference
	Women	0.59 [0.59–0.60]	0.01 * [0.01–0.02]	1.41 [1.39–1.42]	−0.28 * [−0.30–−0.26]
	Children (<15 years)	2.55 [2.53–2.57]	Reference	4.58 [4.53–4.63]	Reference
	Adults (≥15 years)	0.38 [0.38–0.39]	−2.17 * [−2.19–−2.15]	0.93 [0.92–0.94]	−3.65 * [−3.70–−3.60]
	0–4 years	1.12 [1.11–1.14]	Reference	2.39 [2.34–2.44]	Reference
	5–9 years	3.79 [3.75–3.82]	2.67 * [2.63–2.71]	6.90 [6.80–7.01]	4.51 * [4.40–4.62]
	10–14 years	3.02 [2.99–3.06]	1.90 * [1.86–1.94]	5.87 [5.74–6.00]	3.48 * [3.34–3.62]
	15–64 years	0.55 [0.55–0.55]	Reference	1.34 [1.33–1.36]	Reference
	65–74 years	0.09 [0.09–0.09]	−0.46 * [−0.45–−0.47]	0.28 [0.26–0.29]	−1.06 * [−1.08–−1.04]
	≥75 years	0.05 [0.04–0.05]	−0.50 * [−0.49–−0.51]	0.21 [0.20–0.22]	−1.13 * [−1.14–−1.12]
Indicator 6. Percentage of amoxicillin (%)	Total	22.29 [22.28–22.31]		20.46 [20.42–20.64]	
	Men	22.10 [22.08–22.12]	Reference	22.64 [22.58–22.70]	Reference
	Women	22.44 [22.42–22.46]	0.34 * [0.32–0.36]	18.98 [18.93–19.02]	−3.66 * [−3.70–−3.62]
	Children (<15 years)	47.48 [47.43–47.53]	Reference	51.72 [51.60–51.83]	Reference
	Adults (≥15 years)	19.67 [19.65–19.68]	−27.81 * [−27.85–−27.77]	14.45 [14.41–14.48]	−37.27 * [−37.31–−37.23]
	0–4 years	53.07 [52.99–53.15]	Reference	57.92 [57.75–58.09]	Reference
	5–9 years	46.82 [46.73–46.91]	−6.25 * [−6.35–−6.15]	50.65 [50.45–50.85]	−7.27 * [−7.37–−7.17]
	10–14 years	40.85 [40.76–40.94]	−12.22 * [−12.32–−12.12]	37.89 [37.62–38.15]	−20.03 * [−20.13–−19.93]
	15–64 years	22.24 [22.22–22.26]	Reference	16.98 [16.94–17.03]	Reference
	65–74 years	17.85 [17.82–17.88]	−4.39 * [−4.43–−4.35]	12.68 [12.59–12.77]	−4.30 * [−4.36–−4.24]
	≥75 years	12.41 [12.39–12.44]	−9.83 * [−9.87–−9.79]	8.41 [8.35–8.47]	−8.57 * [−8.61–−8.53]
Indicator 7. Percentage of fosfomycin (%)	Total	2.61 [2.60–2.62]		10.47 [10.44–10.50]	
	Men	0.71 [0.70–0.71]	Reference	2.66 [2.64–2.69]	Reference
	Women	4.06 [4.05–4.07]	3.35 * [3.35–3.36]	15.77 [15.72–15.81]	13.11 * [13.06–13.16]
	Children (<15 years)	0.33 [0.32–0.33]	Reference	1.10 [1.08–1.11]	Reference
	Adults (≥15 years)	2.85 [2.84–2.85] *	2.52 * [2.51–2.53]	12.27 [12.24–12.30]	11.17 * [11.12–11.21]
	0–4 years	0.20 [0.19–0.21]	Reference	0.45 [0.43–0.48]	Reference
	5–9 years	0.37 [0.36–0.38]	0.17 * [0.16–0.18]	1.37 [1.32–1.41]	0.92 * [0.86–0.97]
	10–14 years	0.44 [0.43–0.45]	0.24 * [0.22–0.25]	2.27 [2.19–2.35]	1.82 * [1.73–1.90]
	15–64 years	2.32 [2.31–2.32]	Reference	11.04 [11.00–11.08]	Reference
	65–74 years	3.06 [3.05–3.08]	0.74 * [0.73–0.76]	12.14 [12.06–12.26]	1.11 * [1.01–1.20]
	≥75 years	4.47 [4.45–4.48]	2.15 * [2.13–2.17]	15.87 [15.79–15.95]	4.84 * [4.75–4.93]
Indicator 8. Percentage of amoxicillin and beta-lactamase inhibitor (%)	Total	30.36 [30.35–30.37]		20.24 [20.24–20.28]	
	Men	34.02 [33.99–34.04]	Reference	23.96 [23.90–24.03]	Reference
	Women	27.58 [27.56–27.59]	−6.44 * [−6.48–−6.40]	17.72 [17.67–17.76]	−6.25 * [−6.32–−6.17]
	Children (<15 years)	28.28 [28.34–28.33]	Reference	18.65 [18.55–18.74]	Reference
	Adults (≥15 years)	30.58 [30.57–30.60]	2.30 * [2.25–2.35]	20.55 [20.51–20.59]	1.90 * [1.80–2.00]
	0–4 years	26.15 [26.07–26.22]	Reference	16.18 [16.04–16.30]	Reference
	5–9 years	30.06 [29.97–30.14]	3.91 * [3.82, 4.00]	19.48 [19.32–19.64]	3.31 * [3.11–3.51]
	10–14 years	29.08 [28.99–29.17]	2.93 * [2.84, 3.02]	23.38 [23.15–23.62]	7.21 * [6.95−7.47]
	15–64 years	33.88 [33.86–33.90]	Reference	23.76 [23.60–23.81]	Reference
	65–74 years	26.63 [26.59–26.67]	−7.25 * [−7.30–−7.20]	17.14 [17.04–17.24]	−6.62 * [−6.73–−6.50]
	≥75 years	22.44 [22.41–22.47]	−11.44 * [−11.49–−11.39]	13.72 [13.63–13.79]	−10.04 * [−10.14–−9.95]
Indicator 9. Percentage of amoxicillin over the total amoxicillin (plus and without beta-lactamase inhibitor) (%)	Total	42.34 [42.32–42.36]		50.26 [50.19–50.33]	
	Men	39.38 [39.34–39.41]	Reference	48.58 [48.47–48.68]	Reference
	Women	44.87 [44.84–44.90]	5.49 * [5.44–5.54]	51.71 [51.61–51.81]	3.13 * [3.01–3.25]
	Children (<15 years)	62.67 [62.61–62.73]	Reference	73.50 [62.38–62.62]	Reference
	Adults (≥15 years)	39.14 [39.12–39.16]	−23.53 * [−23.62–−23.44]	41.28 [41.20–41.37]	−32.22 * [−32.36–−32.08]
	0–4 years	66.99 [66.90–67.08]	Reference	78.17 [78.00–78.33]	Reference
	5–9 years	60.91 [60.80–61.01]	−6.08 * [−6.23–−5.93]	72.22 [72.00–72.43]	−5.95 * [−6.11–−5.79]
	10–14 years	58.42 [58.30–58.53]	−8.57 * [−8.75–−8.39]	61.84 [61.50–62.18]	−16.33 * [−16.59–−16.07]
	15–64 years	39.62 [39.60–39.65]	Reference	41.69 [41.59–41.79]	Reference
	65–74 years	40.13 [40.06–40.19]	0.51 * [0.45–0.57]	42.53 [42.28–42.77]	0.84 * [0.78–0.90]
	≥75 years	35.61 [35.55–35.67]	4.01 * [−4.07–−3.95]	38.01 [37.78–38.24]	−3.68 * [−3.74–−3.62]
Indicator 10. Percentage of macrolides (%)	Total	12.15 [12.13–12.16]		18.66 [18.62–18.69]	
	Men	11.46 [11.44–11.47]	Reference	19.37 [19.31–19.43]	Reference
	Women	12.68 [12.66–12.69]	1.22 * [1.20–1.24]	18.18 [18.13–18.22]	−1.19 * [−1.23–−1.15]
	Children (<15 years)	11.95 [11.91–11.98]	Reference	16.38 [16.29–16.47]	Reference
	Adults (≥15 years)	12.17 [12.16–12.18]	0.22 * [0.18–0.26]	19.10 [10.06–19.14]	2.72 * [2.66–2.78]
	0–4 years	11.09 [11.04–11.15]	Reference	15.50 [15.38–15.63]	Reference
	5–9 years	11.87 [11.82–11.93]	0.78 * [0.72–0.84]	14.90 [14.76–15.05]	−0.60 * [−0.70–−0.50]
	10–14 years	13.17 [13.10–13.24]	2.08 * [1.99–2.17]	21.35 [21.12–21.57]	5.85 * [5.67–6.03]
	15–64 years	12.58 [12.57–12.60]	Reference	20.24 [20.19–20.29]	Reference
	65–74 years	13.17 [13.14–13.20]	0.59 * [0.53–0.65]	20.45 [20.34–20.55]	0.21 * [0.15–0.27]
	≥75 years	10.06 [10.03–10.08]	−2.52 * [−2.56–−2.48]	14.92 [14.84–14.99]	−5.32 * [−5.38–−5.26]
Indicator 11. Percentage of fluoroquinolones (%)	Total	11.28 [11.27–11.29]		12.95 [12.92–12.99]	
	Men	11.98 [11.96–12.00]	Reference	14.52 [14.47–14.58]	Reference
	Women	10.74 [10.72–10.75]	−1.24 * [−1.26–−1.22]	11.89 [11.84–11.93]	−2.63 * [−2.67–−2.59]
	Children (<15 years)	0.62 [0.61–0.63]	Reference	0.14 [0.13–0.15]	Reference
	Adults (≥15 years)	12.39 [12.38–12.40]	11.77 * [11.74–11.80]	15.42 [15.38–15.46]	15.28 * [15.23–15.33]
	0–4 years	0.62 [0.61–0.63]	Reference	0.06 [0.05–0.06]	Reference
	5–9 years	0.54 [0.53–0.55]	−0.08 * [−0.09–−0.07]	0.08 [0.07–0.10]	0.02 * [0.01–0.03]
	10–14 years	0.71 [0.70–0.72]	0.09 * [0.08–0.10]	0.44 [0.41–0.48]	0.38 * [0.36–0.40]
	15–64 years	8.56 [8.54–8.57]	Reference	11.24 [11.19–11.28]	Reference
	65–74 years	16.60 [16.57–16.63] *	8.04 * [8.00–8.08]	19.99 [19.89–20.10]	8.75 * [8.67–8.83]
	≥75 years	22.09 [22.06–22.13]	13.53 * [13.49–13.57]	24.23 [24.14–24.33]	13.00 * [12.92–13.08]
Indicator 12. Percentage of 3rd generation cephalosporins (%)	Total	2.55 [2.55–2.56]		2.59 [2.58–2.61]	
	Men	2.38 [2.37–2.39]	Reference	2.59 [2.56–2.61]	Reference
	Women	2.68 [2.67–2.69] *	0.30 [0.28–0.32]	2.60 [2.58–2.62] ^NS^	0.01 [−0.01–0.03]
	Children (<15 years)	2.16 [2.14–2.17]	Reference	2.63 [2.59–2.67]	Reference
	Adults (≥15 years)	2.59 [2.59–2.60]	0.43 * [0.41–0.45]	2.58 [2.57–2.60]	−0.05 ** [−0.07–−0.03]
	0–4 years	2.81 [2.78–2.84]	Reference	3.25 [3.19–3.31]	Reference
	5–9 years	2.22 [2.19–2.24]	−0.59 * [−0.62–−0.56]	2.38 [2.32–2.44]	−0.87 * [−0.91–−0.83]
	10–14 years	1.23 [1.21–1.25]	−1.58 * [−1.60–−1.56]	1.52 [1.46–1.59]	−1.73 * [−1.77–−1.69]
	15–64 years	1.99 [1.98–2.00]	Reference	2.08 [2.06–2.10]	Reference
	65–74 years	3.05 [3.03–3.07]	1.06 * [1.03–1.09]	2.89 [2.85–2.94]	0.81 * [0.77–0.85]
	≥75 years	4.27 [4.25–4.28]	2.28 * [2.25–2.31]	3.81 [3.77–3.85]	1.73 * [1.69–1.77]

Data are represented as the percentage with 95%CI in square brackets. ^#^ First-line antibiotics: penicillins with extended spectrum (J01CA), beta-lactamase sensitive penicillins (J01CE), beta-lactamase resistant penicillins (J01CF), and fosfomycin (J01XX01). ^NS^: Not significant. Chi-square test was applied. * *p*-value: <0.001; ** *p*-value: <0.05.

### 2.3. Validation of BIFAP as Source of Data

#### 2.3.1. Representativeness of BIFAP Population Among the Spanish Population

When comparing the BIFAP population with the National Statistics Institute (NSI) population data, we found similarities in terms of age and sex distribution (Table 4). Based on these demographic characteristics, the BIFAP population could be considered representative of the Spanish population.

#### 2.3.2. Comparison of BIFAP and PRAN Data

The indicators that could be compared with PRAN data are presented in Table 5. Other indicators obtained from BIFAP could not be compared, as PRAN does not provide information on the number of dispensed packages, prevalence data, or the number of antibiotic dispensations per patient-year. Additionally, information disaggregated by age or gender was not available for PRAN.

The average antibiotic consumption rate (DID) in the BIFAP population was lower than the national average reported by PRAN for 2018 (10.98 DID versus 15.91 DID) (Table 5). Indicators related to the selection of different antibiotics, measured in DDD, and the percentage of first-line antibiotics closely aligned with those reported by the PRAN for the study period (Table 5). The largest discrepancies were observed in overall antibiotic consumption (DID) and the percentage of amoxicillin over the total amoxicillin (plus and without beta-lactamase inhibitor), where BIFAP values were notably lower than PRAN.

According to the maximum relative difference of 25%, defined as the threshold for acceptable agreement between the two data sources, Indicator 1 exceeded this threshold. Then, it will be excluded from future analyses. Based on the indicators that show good concordance between the two databases (indicators 4 to 12), the overall degree of similarity between them was 8.84% [7.28–10.61], which can be interpreted as the overall level of divergence between the two databases.

## 3. Discussion

This study offers a comprehensive and detailed analysis of antibiotic use within the community setting, leveraging data extracted from BIFAP, a large-scale, population-based Primary Care electronic health records database [15]. Our findings highlight the feasibility and reliability of calculating antibiotic prescribing indicators at the community level using this national electronic database. Moreover, we underscore the significant value of BIFAP in detailing the demographic and clinical characteristics of patients, enabling a more nuanced understanding of prescribing patterns across different age groups and between genders.

The results revealed a prevalence of antibiotic use of 23.3%, which aligns well with the existing literature reporting annual antibiotic prescription rates ranging from 15% to 30% in community settings [5,6,16]. Additionally, data from the Spanish Ministry of Health indicated that 20.5% of the population received at least one antibiotic course in 2021 [17], further corroborating the consistency of our findings with national trends. Notably, 26.6% of antibiotic users were aged 65 years or older. As detailed in Table 1, this group bore a considerable burden of morbidity: 74% of adults and 34% of children had at least one chronic underlying condition, with adults averaging two such conditions. Both groups frequently received concomitant medications. Lifestyle-related risk factors were also prevalent, with 45.7% identified as current or former smokers and approximately 30% reporting alcohol consumption. These findings highlight that a substantial proportion of antibiotic users in the community exhibit characteristics indicative of clinical vulnerability.

Patterns in the selection and use of antibiotic subgroups were broadly consistent with the indicators calculated from BIFAP and those available from PRAN [13] with the exception of the overall antibiotic consumption rate, which was lower than the national estimate reported by PRAN. This supports BIFAP’s potential as a valuable tool for national antimicrobial surveillance. Regarding the overall rate of antibiotic consumption (DID), BIFAP data can be useful for observing the variability across population subgroups. However, although this indicator can be used regionally by the participating regions in BIFAP, it cannot yet serve as a national reference.

Analysis of antibiotic use profile by population subgroups revealed considerable variation by age and gender, with the youngest (0–4 years) and the oldest (≥75 years) patients receiving the most prescriptions. These patterns are consistent with previous European epidemiological studies that emphasize these demographics’ vulnerability to respiratory and urinary tract infections [3,18]. A notable sex disparity was also observed, with women receiving more antibiotics prescriptions than men. This may reflect not only a higher prevalence of infections among women but also differences in healthcare-seeking behavior as women are more frequent users of Primary Care services, potentially contributing to the observed prescription rates [4,19,20].

Despite national guidelines recommending narrow-spectrum antibiotics as first-line agents for conditions commonly treated in Primary Care [12], these accounted for only 26.5% of total DDD and 33.9% of dispensed packages. These data contrast with data from other countries such as Denmark, where antibiotic consumption is primarily based on narrow-spectrum antibiotics and first-line agents, which represent more than 50% prescriptions [21]. The underutilization of first-line agents was particularly pronounced among adults over 75 years (18%), which is concerning given their increased susceptibility to adverse drug events and colonization with resistant organisms [22]. In contrast, non-recommended second-line and third-line antibiotics such as amoxicillin–clavulanic acid, macrolides, fluoroquinolones, and third-generation cephalosporins represented 56.5% of DDD. The high reliance on second-line antibiotics warrants further attention. The excessive and inappropriate use of second-line antibiotics, mainly those with a broad spectrum, can have significant consequences for patients, including increased risk of adverse drug reactions, secondary infections such as *Clostridioides difficile*, and disruption of the normal microbiota. Furthermore, the selective pressure exerted by these agents accelerates the development and spread of antimicrobial resistance, compromising the effectiveness of standard therapies and limiting future treatment options.

These findings are particularly relevant in light of the WHO’s AWaRe classification, which groups antibiotics into Access, Watch, and Reserve categories to guide their appropriate use and minimize the risk of antimicrobial resistance and promotes the preferential use of Access-group antibiotics for common infections due to their lower potential for resistance selection [23]. In this context, the low use of Access-group antibiotics, recommended as first-line options, and the predominance of Watch-group agents such as fluoroquinolones, macrolides, and third-generation cephalosporins in outpatient prescriptions, especially among adults and older patients, are particularly concerning, not only deviating from national guidelines but also contravening international commitments and WHO’s global targets.

These results highlight clear opportunities to improve antibiotic prescribing practices and identify key areas for intervention within Spain’s antimicrobial stewardship initiatives. Efforts should focus on promoting the appropriate use of Access antibiotics while reducing the overuse of broad-spectrum agents, thereby strengthening adherence with both national guidelines and the international recommendations [22,23].

Practical implications. The findings reveal several important implications for optimizing antibiotic prescribing. First, stronger action is needed to enhance compliance with national and international guidelines. Second, interventions should primarily target clinically vulnerable populations, such as very young children and older adults with multiple comorbidities and polypharmacy. Third, the availability of data enabling comprehensive monitoring of antibiotic consumption is crucial for understanding the specific risks of each patient group and identifying key priorities for improvement, thereby supporting the design of targeted interventions and the accurate monitoring of progress over time.

Strengths and limitations. A key strength of the study is the use of a large, individual patient-level database with comprehensive demographic information and linkage to clinical variables, including indications for antibiotic prescriptions, comorbidities and concomitant treatments. These features support more nuanced evaluations of prescribing behavior and risk factors for inappropriate use. The importance of population-based data sources for tracking antibiotic consumption and informing ASP has been previously highlighted [2] since it represents an advantage over aggregated metrics commonly used in large-scale surveillance studies [10,13].

Limitations include the exclusion of hospital prescriptions, lack of data from private healthcare providers, and over-the-counter antibiotic sales, which may lead to underestimation of total antibiotic use. This limitation, noted in similar databases, underscores the importance of integrating multiple data sources to achieve a comprehensive understanding of antibiotic use [2]. Another limitation is the assumption of patient compliance based solely on dispensation records, which may result in overestimation of actual antibiotic consumption [6]. In addition, we point out the use of the DDD for measuring antibiotic consumption in pediatric patients as a limitation, as they are not specifically adjusted for this population. To address this limitation, the number of dispensed packages was also used as an additional measure of antibiotic consumption. Finally, incomplete regional coverage must be considered. In 2018, BIFAP included data from seven regions, making necessary verification of its national representativeness. This is improving as other regions are joining the database. For example, in 2025, BIFAP covered ten regions. As more regions participate, this limitation will be overcome.

Future research: BIFAP enables further in-depth and powerful analysis of antibiotic prescribing given its potential to link data. In this sense, an expanded collection of data linked to diagnosis is currently underway. These data will provide a more comprehensive view of antibiotic consumption in the Spanish community which will strengthen analysis and targeted interventions.

## 4. Materials and Methods

### 4.1. Design

A population-based cross-sectional study was conducted, using individual patients as the unit of analysis. Patients were uniquely identified through their personal social insurance card number. The inclusion criteria comprised all individuals who received at least one prescription for systemic antibacterial agents (ATC code J01, according to the Anatomical Therapeutic Chemical classification system of the World Health Organization, which categorizes medicines based on their therapeutic use) [24], prescribed by the Regional Public Health Services and dispensed by community pharmacies during the study period, from 1 January to 31 December 2018. The reason for using 2018 data for BIFAP validation was based on the fact that this year represented the first period with complete and structured records compiled using standardized dictionaries aligned with the methodological requirements of the study. In addition, 2018 marked the starting point of the time series of the national antibiotic consumption surveillance system which will be established in the following years.

### 4.2. Data Sources and Collection

Patients were identified through BIFAP, a longitudinal population-based electronic database comprising anonymized medical records from patients treated in the Primary Care setting of the Spanish National Health System [15]. The database contained comprehensive information, including individual administrative and sociodemographic data, clinical records, medical diagnoses, interventions, and all medications prescribed by the Primary Care physicians.

Importantly, BIFAP provides more granular information than the aggregated data currently used in PRAN reports, allowing deeper exploration of prescribing patterns by age and gender. Moreover, BIFAP enables the calculation of additional indicators not currently reported by PRAN, such as prevalence of use, number of treatments per patient-year, and indications for which antibiotics were prescribed. In addition, it provided age- and sex-stratified patterns—key metrics for assessing antimicrobial stewardship efforts along with other sociodemographic characteristics of the treated population.

Drug consumption was organized according to ATC codes [24]. Health conditions were recorded as episodes of care using either the International Classification of Primary Care (ICPC) [25] or the International Classification of Diseases (ICD-9) [26]. The computerized system links patient data, medical consultations, and prescriptions enabling the identification of antibiotic prescriptions according to patient age, sex, and conditions for which antibiotics were prescribed. The information contained in the database was anonymized to ensure confidentiality of patients, healthcare professionals, and healthcare centers, in compliance with Spanish and European data protection regulations.

A comparative analysis was conducted between the population included in BIFAP during the study period and the Spanish population reported by NSI [27] to assess similarity in terms of age and sex distribution. The NSI data corresponded to January 2019, reflecting the Spanish demographic structure in 2018.

Validation of BIFAP as a data source for a national surveillance system on antibiotic consumption in the community was carried out by comparing the results of the indicators obtained from BIFAP with those currently monitored by PRAN, using data from the Regional Healthcare Services [13].

At the time of the study, BIFAP collected consultation data from 7566 Primary Care physicians (6419 general practitioners and 1147 pediatricians) across seven Spanish regions [15].

### 4.3. Measurements

#### 4.3.1. Antibiotic Prescribing Indicators

The indicators used in this study were based on those developed by PRAN for monitoring antibiotic consumption in the community setting [11]. The set of indicators included: (a) three quantitative indicators (indicators 1 to 3): overall antibiotic consumption rates (total use of J01), prevalence of antibiotic use, defined as the percentage of individuals in the population (or in the population database BIFAP) who have received at least one course of antibiotics in the year; (b) five indicators related to the selection of antibiotics that should be prioritized as they support stronger evidence, a better safety profile, and alignment with current antimicrobial resistance patterns (indicators 4, 5, 6, 7, 9); (c) four indicators focused on second-line or third-line antibiotics selection or those whose used should be restricted in community settings (indicators 8, 10, 11, 12). The definition and calculation methods for each indicator are provided in Table S1 (Supplementary Data).

These indicators were measured using both DDD and the number of packages, as an approximation of the number of treatment courses prescribed. This dual approach provides a more nuanced assessment of antibiotic use, especially in pediatric populations where DDD is not representative metric.

Drug consumption was measured as the number of DDD prescribed and dispensed by community pharmacies. Given the limitations of the DDD metric, particularly in pediatric populations, indicators were also expressed as the number of packages prescribed and dispensed, serving as an approximation of the number of treatment courses.

#### 4.3.2. Variables Related to Patients

- Administrative data: Dates of patient registration and deregistration from practices, including dates of death.
- Sociodemographic data: Sex and age. Age was categorized into the following groups: 0–4, 5–9, 10–14, 15–64, 65–74, and ≥75 years. Patients under 15 years were classified as pediatric.
- Lifestyle data: Smoking behavior and alcohol intake.
- Concomitant drug treatments
- Concomitant health problems: Underlying chronic health conditions, diagnoses or symptoms that led to patient visits (episodes of care) or reason for antibiotic indication and prescription.
- Death.

### 4.4. Statistical Analysis

A descriptive analysis was performed for patients and antibiotic consumption indicators included in the study. Categorical variables were summarized using absolute and relative frequencies (percentages) with their corresponding 95% confidence intervals (95%CI). Continuous variables with skewed distribution were described using the median (50th percentile) and interquartile range (25th–75th percentiles).

For qualitative indicators, absolute differences with 95% confidence intervals were estimated using a binomial approximation. For quantitative variables the normal approximation was used. All analyses were stratified by sex and age group.

Associations between qualitative variables were assessed using the chi-square (χ^2^) test. For comparisons of antibiotic consumption rates (indicators 1 and 3), Student’s *t*-test and one-way ANOVA were applied as appropriate.

To show the degree of similarity between the BIFAP and PRAN data, absolute and relative differences between the values were calculated. A maximum relative difference of 25% was defined as the threshold for acceptable agreement between the two data sources. This made it possible to determine which indicators would not be appropriated to be calculated using BIFAP.

Based on the indicators that show good concordance between the two databases, the overall level of similarity between them was calculated as the mean of the relative differences (CI95%) as follows: |difference between values|/mean of the values.

Statistical significance was set at a *p* value < 0.05. All statistical analyses were conducted using STATA Corp., version 17.

## 5. Conclusions

This study highlights the value of detailed, patient-level data in monitoring antibiotic use in Primary Care, enabling a more accurate assessment of prescribing practices in the community. These insights are crucial for identifying priority areas and tailoring interventions to promote responsible antibiotic prescribing practices, particularly among vulnerable groups such as young children and older adults. Integrating a comprehensive population database derived from electronic Primary Care records into the PRAN surveillance framework represents a significant step forward. It provides a robust, data-driven foundation to guide targeted policies and actions aimed at optimizing antimicrobial prescribing practices across Spain.

## Figures and Tables

**Table 1:** Sociodemographic and baseline clinical characteristics of the study patients.

Patient’s Characteristics	Patients (Number or Median)	% or [Interquartile Range]
Total	2,191,582	
Pediatric patients (0–14 years), *N* (%)	323,239	14.75
0–4 years	135,716	6.19
5–9 years	109,637	5
10–14 years	77,886	3.55
Adult patients (>15 years), *N* (%)	1,868,343	85.25
15–64 years	1,284,050	58.59
65–74 years	256,778	11.72
≥75 years	327,515	14.94
Age (years), median	47	[26–66]
Pediatric patients	5	[3–9]
Adult patients	52	[37–69]
Sex (*N*, % women)	1,278,559	58.34
Smokers or ex-smokers ^1^	382,659	45.69
Alcohol consumption ^2^	262,549	29.81
Number of comorbidities, median	2	[1–3]
Number of concomitant treatments, median	2	[2–5]
Deaths	12,326	0.56

^1^ Data from 837,436 patients. ^2^ Data from 880,886 patients.

**Table 4:** Structure of BIFAP population compared to the Statistics National Institute population.

	BIFAP Population		NSI * Population		Absolute %-Point Difference NSI *–BIFAP
	N	%	N	%	
Total	9,390,253	100	46,948,740	100	
Gender					
Men	4,575,288	48.72	23,011,370	49.01	0.29
Women	4,814,965	51.28	23,937,375	50.99	−0.29
Age					
<15 years	1,386,847	14.77	6,930,117	14.76	−0.01
0–4 years	465,961	4.96	2,067,503	4.40	−0.56
5–9 years	460,161	4.90	2,356,886	5.02	0.12
10–14 years	460,725	4.91	2,505,728	5.34	0.43
≥15 years	8,003,406	85.23	40,018,623	85.24	0.01
15–64 years	5,990,246	63.79	30,901,366	65.82	2.03
65–74 years	951,954	10.14	4,594,239	9.79	−0.35
≥75 years	1,061,206	11.30	4,523,018	9.63	−1.67

* National Statistics Institute (NSI): data for January 2019 (representative of 2018).

**Table 5:** Comparative data on antibiotic consumption indicators through BIFAP and PRAN *.

Indicators	BIFAP Population Data	PRAN Data *	Absolute Difference BIFAP-PRAN [95%CI]	Relative Difference ^£^ [95%CI]
Indicator 1. Consumption rates of antibiotics for systemic use (J01) (DID: DDD/1000 inhabitants and days)	10.98	15.91	−4.93 [−5.19, −4.67]	−36.67 [−38.63, −34.71]
Indicator 4. Percentage of first-line antibiotics ^#^ (% DDD)	26.52	25.56	0.96 [−0.02, 1.94]	3.69 [1.73, 5.65]
Indicator 5. Percentage of beta-lactamase sensitive penicillins (% DDD)	0.59	0.47	0.12 [−0.86, 1.10]	22.64 [20.68, 24.60]
Indicator 6. Percentage of amoxicillin (% DDD)	22.29	21.69	0.60 [−0.38, 1.58]	2.73 [0.77, 4.69]
Indicator 7. Percentage of fosfomycin (% DDD)	2.61	2.30	0.31 [−0.67, 1.29]	12.63 [10.67, 14.59]
Indicator 8. Percentage of amoxicillin and beta-lactamase inhibitor (% DDD)	30.36	30.07	0.29 [−0.69, 1.27]	0.96 [−1.00, 2.92]
Indicator 9. Percentage of amoxicillin over the total amoxicillin (plus and without beta-lactamase inhibitor) (% DDD)	42.34	49.61	−7.27 [−8.25, −6.29]	−15.81 [−17.77, −13.85]
Indicator 10. Percentage of macrolides (% DDD)	12.15	12.50	−0.35 [−1.33, 0.63]	−2.84 [−4.80, −0.88]
Indicator 11. Percentage of fluoroquinolones (% DDD)	11.28	12.87	−1.59 [−2.57, −0.61]	−13.17 [−15.13, −11.21]
Indicator 12. Percentage of 3rd generation cephalosporins (% DDD)	2.55	2.68	−0.13 [−1.11, 0.85]	−4.97 [−6.93, −3.01]

* Prescriptions from Public Health System in 2018 (without mutual insurance companies) [13]. ^£^ Calculated as: |difference between values|/mean of the values. ^#^ First-line antibiotics: penicillins with extended spectrum (J01CA), beta-lactamase sensitive penicillins (J01CE), beta-lactamase resistant penicillins (J01CF), and fosfomycin (J01XX01).

## Data Availability

The data that support the findings of this study are available upon request from the corresponding authors.

## Supplementary Materials

The following supporting information can be downloaded at: https://www.mdpi.com/article/10.3390/antibiotics14111071/s1, Table S1: Definition and calculation methods for indicators.
