# Supplementary material for: Antibiotic Use in the Community in Spain: A National Surveillance System Within the Framework of the Spanish Action Plan on Antimicrobial Resistance
Source: Antibiotics (Basel). 2025 Oct 24;14(11):1071. doi: 10.3390/antibiotics14111071 (PMC12649326; doi:10.3390/antibiotics14111071)
Supplement: Supplementary file 1 [file antibiotics-14-01071-s001.zip › antibiotics-3907787-supplementary.pdf]

Supplementary Data

Table S1. Quality prescribing indicators: definition, calculation, recommended actions and population to be applied.

Definition	Calculation	Recommended actions	Population to be applied
Indicator 1. Consumption rates of antibiotics for systemic use (J01) (DID: DDD/1000 inhabitants and days)	Number of DDD of antibiotics (J01) x 1000 / number inhabitants (or number of individuals on the database BIFAP) and days	Decrease the total cosumption	Adults and children
Indicator 2. Prevalence of antibiotic use (%)	Number of patients with antibiotic prescriptions (J01) x 100 / number inhabitants (or number of individuals on the database BIFAP)	Decrease the total cosumption	Adults and children
Indicator 3. Total number of antibiotics dispensations per patient-year	Number of antibiotic (J01) dispensations / number of patients with antibiotic (J01) prescriptions	Decrease the total cosumption	Adults and children
Indicator 4. Percentage of first-line antibiotics (%)	Number of DDD or packages of first-line antibiotics* x 100 / total DDD antibiotics (J01)	Prioritize the use of first-line antibiotics over those with higher spectrum	Total population
Indicator 5. Percentage of beta-lactamase sensitive penicillins (%)	Number of DDD or packages of beta-lactamase sensitive penicillins (J01CE) x 100 / total DDD or packages of antibiotics (J01)	Prioritize the use of beta-lactamase sensitive penicillins over antibiotics with higher spectrum	Adults and children
Indicator 6. Percentage of amoxicillin (%)	Number of DDD or packages of amoxicillin (J01CA04) to ≤14 years x 100 / total DDD or packages of antibiotics (J01) to ≤14 years	Prioritize the use of amoxicillin over antibiotics with higher spectrum	Adults and children
Indicator 7. Percentage of fosfomycin (%)	Number of DDD or packages of fosfomycin trometamol (J01XX01) to >14 years x 100 / total DDD or packages of antibiotics (J01) to >14 years	Prioritize the prescription of fosfomycin trometamol versus fluoroquinolones for non-complicated urinary tract infections	Adults
Indicator 8. Percentage of amoxicillin and beta-lactamase inhibitor (%)	Number of DDD or packages of amoxicillin and beta-lactamase inhibitor (J01CR2) to ≤14 years x 100 / total DDD or packages antibiotics (J01) to ≤14 years	Decrease the relative use of beta-lactamase inhibitor	Children
Indicator 9. Percentage of amoxicillin over the total amoxicillin (plus and without beta-lactamase inhibitor) (%)	Number of DDD or packages of amoxicillin (J01CA04) x 100 / [total DDD or packages (amoxicillin (J01CA04) + amoxicillin and beta-lactamase inhibitor (J01CR02)]	Prioritize the use of amoxicillin over amoxicillin with beta-lactamase inhibitor	Adults and children

Indicator 10. Percentage of macrolides (%)	Number of DDD or packages of macrolides (J01FA) x 100 / total DDD or packages of antibiotics (J01)	Decrease the relative use of macrolides	Adults and children
Indicator 11. Percentage of fluoroquinolones (%)	Number of DDD or packages of fluoroquinolones (J01MA) to >14 years x 100 / total DDD or packages of antibiotics (J01) to >14 years	Decrease the relative use of fluoroquinolones	Adults
Indicator 12. Percentage of 3rd generation cephalosporins (%)	Number of DDD or packages of 3rd generation cephalosporins (J01DD) x 100 / total DDD or packages of antibiotics (J01)	Decrease the relative use of 3rd generation cephalosporins	Adults and children

* First-line antibiotics: penicillins with extended spectrum (J01CA), beta-lactamase sensitive penicillins (J01CE), beta-lactamase resistant penicillins (J01CF), and fosfomycin (J01XX 01)
